# Dr. Johann Michael Robert Amthor

**Published:** 1897-06

**Authors:** Elias C. Price

**Affiliations:** 1012 Madison Avenue; Baltimore


### DR. JOHANN MICHAEL ROBERT AMTHOR.

1012 Madison Avenue.

BALTIMORE, May 26th, 1897.

DR. C. B. KNERR, 1137 Spruce Street, Philadelphia, Pa.

DEAR DOCTOR.—I write to inform you of the death of one of Dr. Hering's old friends, Johann Michael Robert Amthor, M. D. He died May 19th, 1897, of hypertrophy with dilatation of the heart and nephrolithasis, aged seventy-six years, one month, nineteen days, at his residence, 427 North Broadway, Baltimore, Md.

He was born in Gotha, Germany, March 4th, 1821. He was educated at the Gymnasium Ernestinum of that city; pursued his medical studies under the direction of three eminent homeopathic physicians, Dr. Blau, Dr. Plaubel, and Dr. Wohlgemuth. In 1852 he came to America and settled in Baltimore. Just before leaving Germany he married Fredericke Oschman, also of Gotha; they had four sons and two daughters; all their children are dead except one daughter; his wife died about three months ago.

I do not know whether you are an anti-vaccinationist, but three of his sons died of small-pox, he having neglected to vaccinate them, one of them, Dr. Robert Amthor, Jr.

Yours fraternally, ELIAS C. PRICE.

